# An interpretable crop leaf disease and pest identification model based on prototypical part network and contrastive learning

**DOI:** 10.1038/s41598-025-22521-1

**Published:** 2025-11-04

**Authors:** Bingjing Jia, Jinyu Zeng, Zhiwei Zheng, Hua Ge, Chenguang Song

**Affiliations:** 1 College of information & Network Engineering, Anhui Science and Technology University, Bengbu, 233000 China; 2 College of Intelligent Manufacturing, Anhui Science and Technology University, Chuzhou, 239000 China

**Keywords:** Crop leaf disease and pest identification, Interpretability, CPNet, Contrastive learning, Machine learning, Classification and taxonomy

## Abstract

The disease and pest recognition algorithms based on computer vision can automatically process and analyze a large amount of disease and pest images, thereby achieving rapid and accurate identification of disease and pest categories on crop leaves. Currently, most studies use deep learning models for feature extraction and identification of crop leaf disease and pest images. However, these methods are often seen as “black box” model, making it difficult to interpret the basis for their specific decisions. To address this issue, we propose an intrinsically interpretable crop leaf disease and pest identification model named **C**ontrastive **P**rototypical Part **Net**work (CPNet). The idea of CPNet is to find the key regions that influence the model’s decision by calculating the similarity values between the convolutional feature maps and the learnable latent prototype feature representations. Moreover, because the limited availability of data resources for crop leaf disease and pest images, we employ a supervised contrastive learning strategy to capture the similar information between examples in one class and contrast them with examples in other classes. Finally, we evaluate our approach on four publicly available datasets, and the experimental results demonstrate that our proposed CPNet not only achieves improvements in performance over baseline methods across multiple datasets, but also provides interpretable evidence for crop leaf disease and pest identification.

## Introduction

Crop diseases and pests are one of the major disasters restricting agricultural production, seriously affecting the yield and quality of crops^1^. The occurrence of disastrous crop diseases and pests will aggravate the shortage of food supply^2^. The prevention and control of crop diseases and pests play a very important role in agricultural production, and the accurate identification of crop leaf diseases and pests is an important part of crop disease and pest control work^3^.

With the rapid development of computer vision, machine learning has gradually been introduced into the identification of crop leaf diseases and pests^4^. The theory of machine learning mainly designs and analyzes algorithms that enable computers to “learn” automatically^5^. These algorithms can automatically analyze and obtain rules from data, and use the rules to predict data^6^. However, the early machine learning models used in the crop leaf disease and pest recognition mostly leverage the image processing techniques ormanually designed features^7^. These algorithms can only extract low-level features, making it difficult to extract deep and complex image feature information^8^. That is, they cannot fully capture the complexity of the data, thus affecting the accuracy of the identification of the leaf area where the diseases and pests are located^9^.

In recent years, as an important branch of machine learning, deep learning has be come a popular technology in various fields such as disease diagnosis^10^, dialogue system^11^, and product recommendation^12^. To date, deep learning models have been successfully applied in agricultural studies in part because they can automatically extract the characteristics of the target area of diseases and pests from a large number of crop leaf images, thereby replacing the traditional recognition methods that rely on manual feature extraction^13^. Deep learning can automatically, efficiently, and accurately extract the characteristics of the target area of diseases and pests from a large number of crop leaf images, thereby replacing the traditional recognition methods that rely on manual feature extraction^14^. It is composed of multiple-layered neural networks, and has good autonomous learning ability and feature expression ability, automatically extracting the image characteristics of leaf diseases and pests to achieve image classification and recognition^15^.

Deep learning constructs a multi-level neural network model, which realizes the understanding of the deep semantic features of the data through layer-by-layer feature extraction and abstraction^16^. However, with the construction of multi-layer neural networks, each layer generates a large number of parameters and nonlinear activation functions^17^. Although this kind of architecture performs well in dealing with complex data and tasks, its high complexity and nonlinearity lead to low transparency and poor interpretability. It is difficult for users to understand the decision basis of the model, doubt its credibility, and regard the deep model as a data-driven black box^18^. In the existing deep learning-based crop leaf disease and pest recognition methods, although the model gives the predicted category of diseases and pests, it does not explain the decision-making process and attribution of the model to researchers, which will reduce their trust in the final prediction results^19^.

In fact, improving the interpretability of crop leaf disease and pest recognition models can not only increase the transparency of themodels but also help researchers understand the basic principles of model decision-making, strengthen the trust be tweenmodels and researchers, and increase social acceptance^20^. Therefore, in this paper, we develop an intrinsically interpretable crop leaf disease and pest recognition model named **C**ontrastive **P**rototypical Part **Net**work (CPNet). First, to identify the categories of pests and diseases on crop leaves while finding the infected areas that influence the model’s decisions, we use the Prototypical Part Network (ProtoPNet)^21^ to calculate the similarity values between the convolutional feature maps of the images and the latent prototype feature representations. Generally, a higher similarity score denotes to what extent the region has a greater impact on the model’s decision. Second, in practice, some crop leaf diseases and pests have low incidence and high cost of disease and pest image acquisition, resulting in only a few or dozen training data collected^22^. To solve this problem, we introduce supervised contrastive learning strategy^23^, which effectively captures similar features among instances of the same category and differentiates features between instances of different categories in crop leaf disease and pest images. In summary, the contributions of this paper include the following several aspects:

In this paper, we study a novel task of interpretable crop leaf disease and pest identification from the perspective of image classification.

We propose an intrinsically interpretable model for crop leaf disease and pest recognition. CPNet not only can automatically find key regions of disease and pest images that influence model decisions, but also achieve improvements in performance by learning both similarities within intra-class leaf disease and pest images and differences within intra-class leaf disease and pest images.

To the best of our knowledge, CPNet is the first prototype learning based crop leaf disease and pest identification model in agricultural research domain.

We conduct extensive experiments on four real-world datasets, and experimental results show that CPNet presents competitive predictive performance and provides reliable interpretable evidence for crop leaf disease and pest recognition.

The rest of the paper is organized as follows: Section. 1 “Related work” reviews the related work on crop leaf disease and pest identification. Section. 2 “Materials and methods” describes the datasets and introduces the details of the proposed CPNet framework. In section. 3 “Results and analysis”, we first baseline methods, and then present the experimental results of CPNet. Section. 4 “Conclusion” concludes this work and discuss directions for future work.

## Related work

The identification of crop leaf diseases and pests aims to recognize the types of diseases and pests on crop leaves rapidly and accurately, enabling the adoption of corresponding prevention and control measures^24^. Currently, there are numerous literature in the identification of crop leaf diseases and pests, and we will briefly review studies on this topic based on machine learning and deep learning.

### Crop leaf disease and pest identification based on machine learning

In the early stages of digital agriculture, research on the identification of crop leaf diseases and pests primarily relied on traditional machine learning image recognition algorithms^25^. These works involved hand-crafted feature extraction from leaf images. The features were utilized to train shallow classifier models such as Decision Tree (DT)^26^, Naïve Bayes (NB)^27^, Support Vector Machines (SVM)^28^, Simple Linear Iterative Clustering (SLIC)^29^ and K-Means Clustering (KMeans)^30^. For instance, Rajesh et al.^31^ proposed the use of DT to identify and classify leaf diseases, enhancing disease detection accuracy within a shorter-time frame. Javidan et al.^32^ employed KMeans technology to locate infected areas in images, which were then classified by using SVM. The feature extraction in traditional machine learning primarily relied on manual efforts, necessitating expert experience for feature selection and extraction. However, this process was lanbor-intensive, time-consuming and in some cases impractical^33^. It was noteworthy that those methods may lead to several challenges, including the curse of dimensionality, difficulties in modeling nonlinear data, limited generalization ability, and ineffectiveness in handling large-scale data^34^. In recent years, deep learning models combined with computer vision technology have been widely applied in crop disease and pest identification, significantly advancing the automation of crop disease and pest recognition^35^. Deep learning reduces the need for manual feature engineering by automatically learning feature representations from raw data through multi-layer network structures^39^. Many researchers have proposed crop leaf disease and pest identification models based on various deep learning algorithms, efficiently addressing crop leaf disease and pest identification issues^37,38^. Most of these models primarily rely on Convolutional Neural Networks (CNNs) and attention mechanisms for the identification of crop leaf diseases and pests^39^.

Convolutional Neural Network^40^, a deep learning architecture, is particularly suitable for image processing and computer vision tasks due to its design principles that excel in handling data with grid structures, such as images. The deep network structure of CNN enables it to learn more complex and abstract features like shapes, textures, and intricate patterns, significantly enhancing recognition accuracy compared with traditional methods^41^. Chen et al.^42^ proposed a method based on a deep convolutional network for citrus leaf disease and pest identification, which augmented the LeNet architecture by increasing the number of layers, resulting in a seven-layer network structure. However, merely increasing the number of layers did not significantly enhance the recognition effect. To learn deeper features and improve the recognition accuracy of the model, Yu et al.^43^ proposed an optimized deep residual network system for tomato pest diagnosis. This system not only improves ResNet50 but also introduces the Fruit Fly Optimization Algorithm (FOA), enabling effective diagnosis of tomato pests. Experimental results show that the average accuracy of this model is 94.4%. CNN utilizes local receptive fields and parameter sharing, progressively expanding the receptive field through multi-layer stacking to achieve global modeling. CNN is more direct and efficient in global feature extraction. However, due to its fixed-size convolutional kernels and pooling operations, CNN’s ability to capture subtle local variations is relatively limited^44^.

Traditional CNNs, constrained by the local receptive field of convolution operations, tend to obtain local optimal solutions, leading to the loss of feature information. Consequently, the attention mechanism^45^, which selectively focuses on informative features of interest, has been extensively researched. Inspired by human visual attention characteristics, the attention mechanism aims to enable models to automatically learn and concentrate on crucial parts of input data. In contrast to CNN’s global feature extraction, the attention mechanism specializes in precise representation of local information. For instance, in image processing tasks, the attention mechanism can emphasize key information and suppress unimportant details by assigning different weights to features in different regions^46^. Wu et al.^47^ proposed an improved model for woody fruit plant leaf disease recognition based on ResNet101, incorporating the SENet attention mechanism to enhance the model’s feature extraction capabilities. However, the recognition accuracy remained relatively low. To further strengthen the focus on target regions and improve recognition performance, Haider et al.^48^ introduced three self-attention RBNet architectures. These models first extract image features, then select the optimal features through dragonfly optimization and utilize Bayesian optimization for hyperparameters. The proposed methods achieved accuracies of 98.60% and 93.90%, respectively. The attention mechanism possesses powerful long-range modeling capabilities, which can complement CNNs. Some scholars have proposed models that integrate CNNs with attention mechanisms. Nawaz et al.^49^ introduced the CoffeeNet model, which leverages an enhanced keypoint estimation framework, ResNet-50, and an attention mechanism to extract features of various coffee plant leaf diseases.

### Crop leaf disease and pest recognition based on interpretability

Deep learning simulates the learning process of the human brain through a multi-layered neural network structure, automatically extracting features from data and performing high-level abstract representations, thereby enabling accurate image recognition and classification^50^. However, due to their high nonlinearity and end-to-end complexity, most popular deep learning-based image recognition models are difficult to understand and interpret, making it challenging for users to comprehend the basis of the model’s decisions^51^. Consequently, researching the interpretability of models can assist researchers in understanding the working principles of the models, breaking the “black box” of neural networks, and providing human-understandable explanations for the decisions made by the algorithms^52^. Based on various criteria for classifying machine learning interpretability methods, interpretability research can be divided into two categories: Post-hoc Interpretation Methods and Intrinsically Interpretable Models.

**Post-hoc Interpretation Methods.** Post-hoc Interpretation Methods^53^ focuses on black-box models, leveraging algorithms like visualization analysis, importance analysis, and other means to analyze the model and infer its decision-making process. Examples include feature attribution^54^, permutation importance^55^, and class activation mapping^56^. For instance, Coulibaly et al.^57^ utilized Convolutional Neural Networks to identify and locate pests in crops, and employed Grad-CAM and LIME to evaluate the interpretability of the proposed model. Ethiraj et al.^58^ introduced DNet-SVM for early recognition of sugarcane diseases and pests, and utilized LIME to explain the reasons behind classifying images as healthy or diseased. Similarly, Hernández et al.^59^ used grapevine downy mildew as an example, while Natarajan et al.^60^ employed plant diseases as a case study, to detect plant diseases symptoms. They also employed the Grad-CAM method to visualize the infected areas on plant leaves, explaining the attribution of neural networks in leaf disease detection. Furthermore, Dai et al.^61^ proposed DFN-PSAN for plant disease and pest recognition. This model uses YOLOv5 as the overall architecture and designs a novel PSAN classification network structure to recognize plant diseases and pests in natural field environments. Additionally, it adopts the t-SNE method and SHAP explainable AI (XAI) visualization methods to interpret whether the model focuses on the characteristics of plant diseases and pests.

**Intrinsically Interpretable Models.** Intrinsically Interpretable Models^62,63^ require us to select human-understandable features and employ inherently interpretable models during the problem-solving process, such as rule-based models and linear models. Our model belongs to the category of Intrinsically Interpretable Models. This model is a self-explanatory one that integrates interpretability into its specific structure, enabling the model itself to possess interpretability. The model outputs both the result and the reasons behind it, thereby ensuring the reliability and safety of the interpretation results. CPNet can find key areas that influence the model’s decisions and predict the categories of diseases and pests by calculating the similarity values between the convolutional feature maps of images and the latent prototype features. This approach explains the decision-making process and attribution of the model, thereby enabling interpretable image classification and recognition of crop leaf diseases and pests.

## Materials and methods

### Datasets

The Wheat Plant Diseases dataset is released by Kushagra et al^64^. This dataset is designed to empower researchers and developers in creating robust machine learning models for classifying various wheat plant diseases. It offers a collection of high-resolution images showcasing real-world wheat diseases without the use of artificial augmentation techniques. Since our task focuses on the identification of crop leaf diseases and pests, we conducted rigorous data screening on the dataset under the guidance of relevant agricultural experts. Specifically, through careful comparison and analysis, we removed duplicate images and incorrectly classified images from the original public dataset, and deleted disease categories unrelated to leaf diseases and pests, such as Root Rot and Fusarium Head Blight. Table1 shows the detailed data of various samples in the Wheat Plant Diseases dataset actually used in the experiment.

**Table 1:** Detailed descriptions of the various types of samples within the Wheat Plant Diseases dataset.

Dataset	Category	Number
Wheat Plant Diseases	Healthy	561
	Rust	686
	Mildew	268
	Gibberellins	384
	Leaf blight	360
	Aphid	852
	Mite	587
	Stem fly	226

Dataset for Crop Pest and Disease Detection is developed by Mensah Kwabena et al.^65^. This dataset was collected on local farms in Ghana using high-resolution camera device. The images within the dataset were captured under various conditions and with different backgrounds such as white, dark, illuminated, and real backgrounds. The original .jpg images were in varied dimensions, namely: (400$\times$400), (487$\times$1080), (1080$\times$518), (3024$\times$4032), and (4032$\times$3024). The Crop Pest and Disease Detection dataset includes pest and disease data for Maize, Cashew , Cassava, and Tomato. For our experiments, we have selected the first three types of crop leaf diseases and pests and divided them into three separate datasets. These three datasets are named as Maize Leaf Disease and Pest dataset, Cashew Leaf Disease and Pest dataset, and Cassava Leaf Disease and Pest dataset. The detailed data of various samples in these three datasets are shown in Table 2.

**Table 2:** Detailed descriptions of the various types of samples comprising the Maize, Cashew, and Cassava Leaf Disease and Pest datasets.

Dataset	Category	Number
Maize Leaf Disease and Pest	Grasshopper	673
	Leaf Beetle	934
	Leaf Blight	995
	Leaf Spot	1244
	Streak Virus	924
Cashew Leaf Disease and Pest	Anthracnose	1720
	Healthy	1275
	Leaf Miner	1378
	Red Rust	1682
Cassava Leaf Disease and Pest	Bacterial blight	2614
	Brown Spot	1481
	Green Mite	1015
	Healthy	1193
	Mosaic	1205

The images of all four datasets were captured in natural environments and saved in JPG format, with each image resized to 224 $\times$ 224 pixels. The images have only category labels and no content annotations. Furthermore, the classification model splits the datasets into training and test sets with a ratio of 80% and 20% respectively. Throughout the experiment, due to the presence of fewer samples and class imbalance issues in different datasets, we employed a 10-fold offline data augmentation to reduce overfitting on specific datasets and enhance the model’s stability and generalization capabilities in real-world applications. The data augmentation methods we used include skew, shear, distortion, left-right flip, and color enhancement. The specific parameters were optimized through grid search as follows: skew: uniformly sampled within the [−45$^{\circ }$,+45$^{\circ }$] range to avoid directional bias; shear: maximum angle of 10$^{\circ }$ to maintain the integrity of the lesion; distortion: intensity coefficient of 5 to simulate natural deformation; left-right flip: applied with a 50% probability to account for uncertainty in the shooting orientation; color enhancement: random shifts in the HSV color space, with hue ±0.1, saturation ±0.3, and brightness ±0.2. This is particularly targeted at color-sensitive categories (e.g., Brown Spot and Green Mite) to ensure that the enhanced images retain key color features. All enhancements are implemented using OpenCV, ensuring that the pixel values are normalized to the [0,1] range after transformation.

### Problem formulation

Currently, the task of crop leaf disease and pest recognition involves assigning corresponding labels to images from a given set of categories for classification and identification. A common research approach is to perform feature extraction and recognition of crop leaf diseases and pests based on deep learning. However, the research work presented in this paper focuses on interpretable crop leaf disease and pest recognition by prototypical part network and contrastive learning. Given an image $\textrm{x}$ of crop leaf disease and pest, with its associated category label $\textrm{y}\in \{0,...,c,...,C\}$, the model learns a mapping function $\mathscr {F}{:}\mathscr {F}(\textrm{x})\rightarrow \hat{\textrm{y}}$ that can predict the category of the given image $\textrm{x}$, where $\hat{\textrm{y}}$ is the predicted probability of the leaf disease and pest image $\textrm{x}$ belonging to a certain category. The goal of this research is to optimize the mapping function $\mathscr {F}$ to maximize prediction probability and automatically identify the infected areas of crop leaf diseases and pests, providing interpretable evidence for the final classification results.

### CPNet network architecture

**Figure 1:**
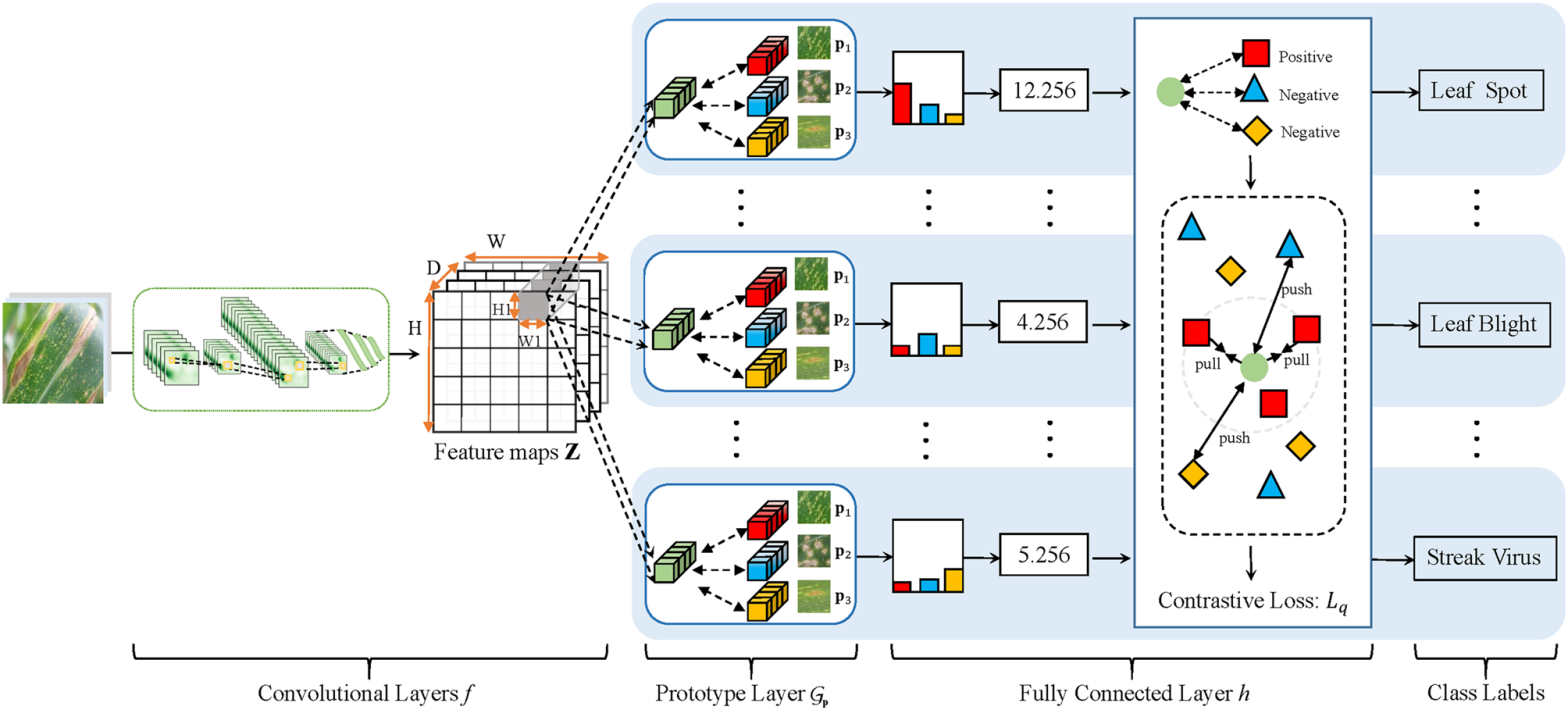
The proposed model framework.

The overall architecture of the CPNet is shown in Fig. 1. CPNet is mainly composed of convolutional layers, a prototype layer, and a fully connected layer. The input is an image $\textrm{x}$. The output is the predicted probability distribution and corresponding interpretable evidence for the given image $\textrm{x}$. First, the model uses the convolutional layer *f* to extract a meaning feature representation $\textbf{Z} = f(\textrm{x}) \in \mathbb {R}^{H \times W \times D}$ from the input image $\textrm{x}$. Second, for each prototype, the prototype layer $\mathcal {G}_{\textbf{p}}$ computes a similarity matrix $\textbf{M}^{\textrm{x}}_{\textbf{p}_{i}} \in \mathbb {R}^{H \times W }$ between the convolutional feature maps $\textbf{Z}$ and a learnable latent prototype feature representation $\textbf{p}_{i} \in \mathbb {R}^{1 \times 1 \times D}$. A similarity score from $\textbf{M}^{\textrm{x}}_{\textbf{p}_{i}}$ indicates that to what extent prototype $\textbf{p}_{i}$ is present in a particular region of image $\textrm{x}$. If there are *m* prototype vectors (i.e., $\textbf{P} = [\textbf{p}_{1}, \cdots , \textbf{p}_{i}, \cdots , \textbf{p}_{m}] \in \mathbb {R}^{m \times 1 \times 1 \times D}$), we can calculate their corresponding similarity matrices (i.e., $\{\textbf{M}^{\textrm{x}}_{\textbf{p}_{1}},\cdots , \textbf{M}^{\textrm{x}}_{\textbf{p}_{i}}, \cdots , \textbf{M}^{\textrm{x}}_{\textbf{p}_{m}} \}$). Then, the prototype layer $\mathcal {G}_{\textbf{p}}$ selects the maximum value in each similarity matrix $\textbf{M}^{\textrm{x}}_{\textbf{p}_{i}}$ as its output (i.e., $\textbf{s} = [s_{1}, \cdots , s_{i}, \cdots , s_{m}] \in \mathbb {R}^{m}$). A maximum similarity value denotes corresponding region of image $\textrm{x}$ that is the most similar part to the learned prototype $\textbf{p}_{i}$. Next, the similarity vector $\textbf{s}$ produced by the prototype layer $\mathcal {G}_{\textbf{p}}$ is fed into the fully connected layer to generate the final classification logits. These logits are normalized using the softmax function to obtain the predicted probability distribution of disease and pest categories. In addition, CPNet employs supervised contrastive learning strategy to map examples of each class together and to separate them with the examples from other classes in the latent feature representation space. It aims to find the similarities of intra-class leaf disease and pest images and differences of intra-class leaf disease and pest images, improving the overall discriminative capability of the model.

#### Convolution layer

The convolutional layers extract local features from images through convolution operations, automatically learning and recognizing specific patterns in images such as edges, textures, and shapes. Effective recognition of crop leaf diseases and pests, therefore, begins with extracting features from these local regions. The convolutional layer aims to produce a raw feature representation for the image. The convolutional layers *f* first are borrowed from the traditional network(e.g. VGG19, ResNet152, DenseNet161) for feature extraction. The main different is that extra two 1$\times$1 convolutional layers are added to adjust the number of channels for top-level feature map. We use ReLU as the activation function for all convolutional layers except the last for whcih we use the sigmoid activation function. Given an input image $\textrm{x}$, we convert the image into a sequence of feature vectors:

1 $$\begin{aligned} {[}\textbf{z}_{1},\ldots ,\textbf{z}_{i},\ldots ,\textbf{z}_{m}]=\textrm{Conv2D}(\textrm{x})\in \mathbb {R}^{H\times W\times D} \end{aligned}$$

where *H* and *W* are the height and width of representation obtained at the last convolutional layer for image $\textrm{x}$, and *D* is the number of channels in this layer.

#### Prototype layer

The fundamental idea of the prototype layer $\mathcal {G}_{\textbf{p}}$ is to select highly interpretable (i.e., representative) prototypical part by computing the similarity values $\textbf{s}$ between the convolutional feature maps $\textbf{Z}$ of a input image $\textrm{x}$ and the latent prototype feature representations $\textbf{P}$. The prototypical part represent the key region that influence the model’s decision-making. Since the depth of each learned prototypical part is the same as the depth of the convolutional output, but the height and width of each prototypical part are smaller than those of the entire convolutional output, each prototype will be used to represent a prototypical activation pattern in a feature map of the convolutional output, which in turn corresponds to a feature region of a training image in the original pixel space. Therefore, in this work, each prototype can be understood as the latent representation of some prototypical parts of the training images.

Given a convolutional output feature vector $\textbf{z} \in f(\textrm{x})$, and the *m*-th prototype unit $\mathcal {G}_{\textbf{p}_m}$ in the prototype layer $\mathcal {G}_{\textbf{p}}$ computes the Euclidean distances between the *m*-th prototypical part $\textbf{p}_m$ and all the feature maps in $\textbf{Z}$ that have the same shape as $\textbf{p}_m$. Formally, the prototype unit $\mathcal {G}_{\textbf{p}_m}$ is formulated as follows.

2 $$\begin{aligned} \mathcal {G}_{\textbf{p}_m}(\textbf{z})=max_{\tilde{\textbf{z}}\in \text {feature maps}(\textbf{Z})}\log \Bigg (\frac{\Vert \tilde{\textbf{z}}-\textbf{p}_m\Vert _{2}^{2}+1}{\Vert \tilde{\textbf{z}}-\textbf{p}_m\Vert _{2}^{2}+\varepsilon }\Bigg ) \end{aligned}$$

where $\tilde{\textbf{z}}\in \text {feature maps}(\textbf{Z})$ is the nearest latent feature map. Then, the Euclidean distance is inverted into similarity scores $\textbf{s}$ using Eq. (2). The similarity scores for each category is visualized as the corresponding activation map, where the maximum similarity value indicates the strength of the prototypical part in the activation map. This activation map preserves the spatial relation of the convolutional outputs, and can be upsampled to the size of the input image to produce a heat map that identifies which part of the input image is most similar to the learned prototypical part. Next, global max pooling is used to select the maximum similarity value among the activation maps of similarity scores produced by each prototype unit $\mathcal {G}_{\textbf{p}_m}$. This can be understood as the most similar part between the learned prototype $\textbf{p}_m$ and a certain convolutional feature map of the input image $\textrm{x}$. In the model, in order to keep each category represented by a certain number of prototypical part, each of our categories is randomly assigned with 10 prototype images for spatial Euclidean distance computation.

**Figure 2:**
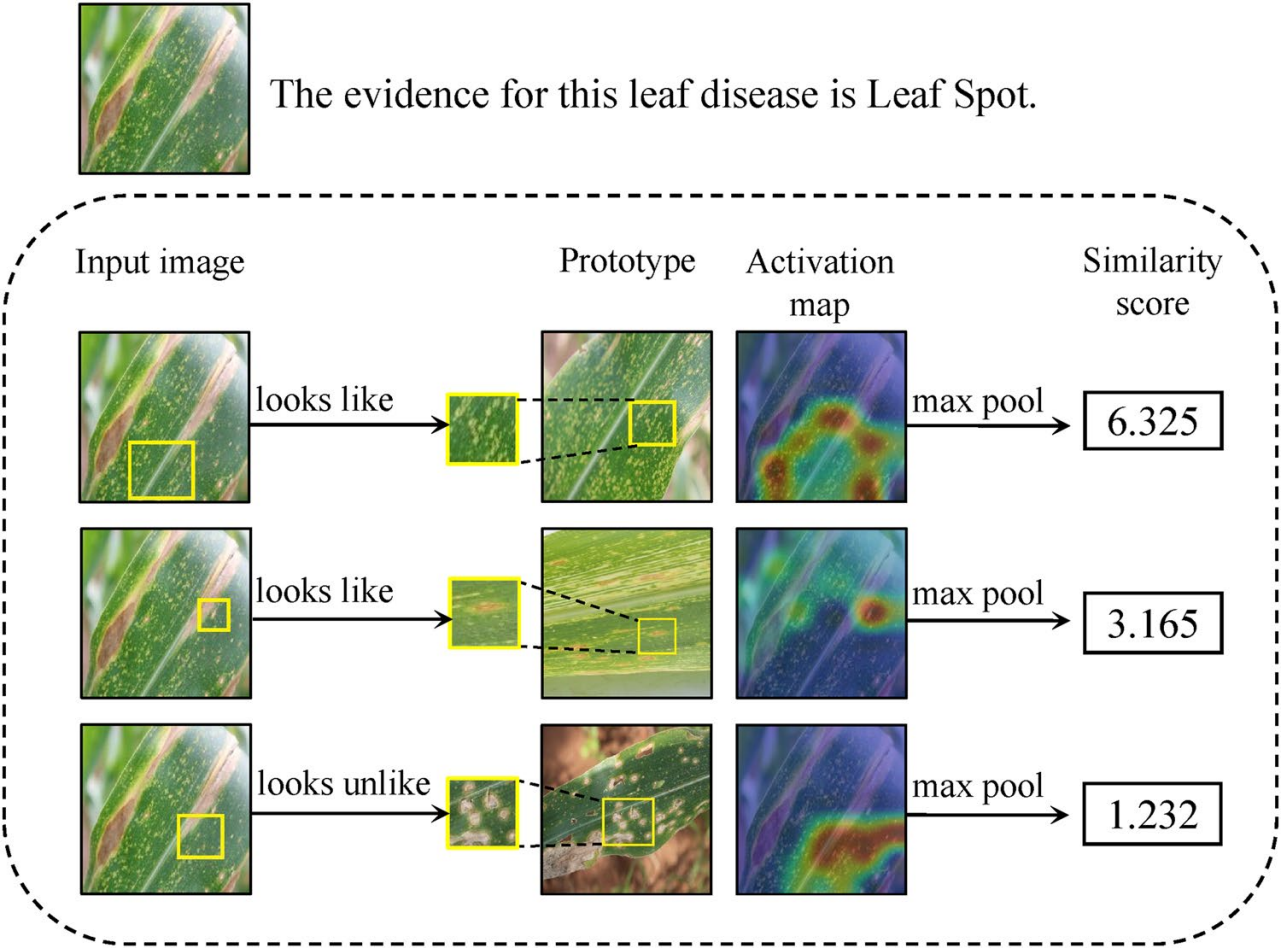
Reasoning process of how CPNet identify a input image of the Leaf Spot disease as the correct class.

Figure 2 shows the classification of Maize Leaf Spot is taken as an example to demonstrate its inference process based on the prototypical part. The prototype layer obtains the similarity scores between the convolutional feature maps of the test image and the prototypical part by calculating the Euclidean distance between them, and visualizes the related activation graphs based on the similarity scores of each category, and then extracts the maximum value from each channel of the activation graphs using global maximum pooling to produce the maximum similarity score for each category. In this case, the closer the Euclidean distance, the higher the similarity score will be, and the greater the influence on the model’s final decision (as exemplified by the first two rows in Fig. 2). Conversely, the farther the Euclidean distance, the lower the similarity score (as exemplified by the last row in Fig. 2), and the lesser the influence on the model’s final decision.

#### Fully connected layer

The fully connected layer integrates and abstracts the features learned in the previous layers for use in the execution of classification or regression tasks. The fully connected layer linearly transforms the input data by means of a weight matrix and a bias vector. The fully connected layer of the CPNet model multiplies the similarity scores generated by the prototype layer $\mathcal {G}_{\textbf{p}}$ with the weight matrix $\textbf{W}_h$ in the fully connected layer, and then feeds the results into a softmax layer for normalization processing. Finally, it produces the prediction result for the given leaf disease and pest image. Therefore, the leaf disease and pest images predictor is defined as:

3 $$\begin{aligned} \widehat{\varvec{\textbf{y}}}=\textrm{Softmax}(\textbf{R}_{zg}\textbf{W}_h+\textbf{b}) \end{aligned}$$

where $\textbf{W}_h\in \mathbb {R}^{d\times C}$ is the learned transformation matrix, $\textbf{R}_{zg}$ is visual feature representation, $\textbf{b}$ is a bias term, and $\widehat{\varvec{\textbf{y}}}=[\hat{\textrm{y}}_0,...,\hat{\textrm{y}}_c,...,\hat{\textrm{y}}_C]$. $\hat{\textrm{y}}_{c}$ denote the predicted probability that the input image belongs to the *c*-th class. Thus, for a given image *x*, we can define the cross-entropy loss function as follows.

4 $$\begin{aligned} \mathscr {C}_{ce}(\Theta )=-\frac{1}{N}\sum _{n=1}^N\sum _{c=1}^C\textrm{y}_c^{(n)}\log (\widehat{\textrm{y}}_c^{(n)}) \end{aligned}$$

where $\Theta$ is the learned parameters of the model, *N* is the number of training samples, *C* is the number of classes, $\textrm{y}_c^{(n)}$ is the probability that the *n*-th sample has the true label in the *c*-th class, and $\widehat{\textrm{y}}_c^{(n)}$ is the probability that the *n*-th sample is predicted to be in the *c*-th class.

#### Model learning

**Figure 3:**
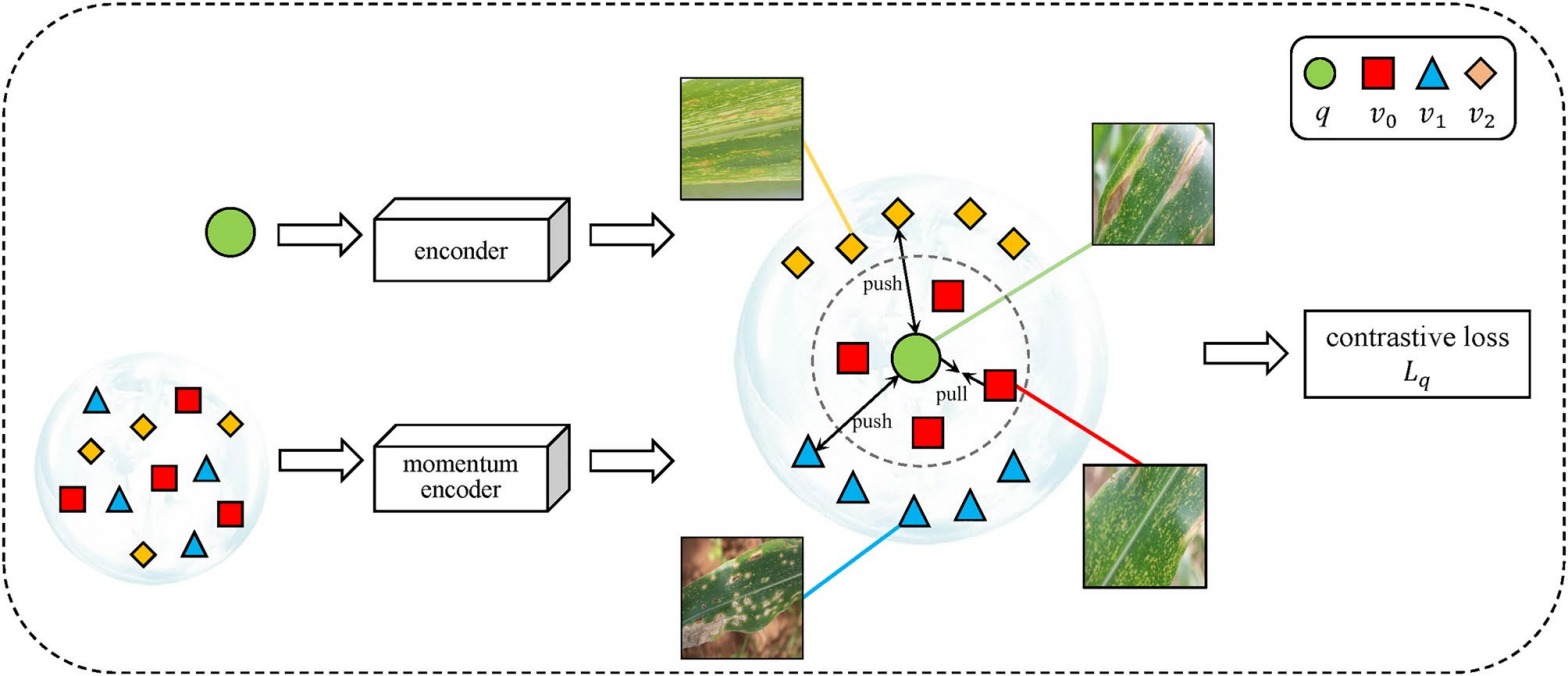
Schematic diagram of contrastive learning structure.

Contrastive learning refers to learning the common features among instances of the same category and distinguishing the differences between instances of different categories. It can be viewed as a dictionary query task, i.e., the task of training an encoder so as to do a dictionary query. Assuming that there is already an encoded key value *q* (a feature), and a series of encoded samples $\{v_0,...,v_k,...,v_K\}$, then, $v_k$ can be viewed as the key values in the dictionary. Assuming that there is only one key value $v_+$ in the dictionary i.e., it matches *q*, then *q* and $v_+$ are positive samples of each other, and the rest of the key values are negative samples of *q*. Once the pairs of positive and negative samples are defined, a loss function for contrastive learning is needed to guide the model to learn. This loss function needs to fulfill these requirements, i.e., the value of this loss should be lower when the key value *q* is similar to the only positive sample $v_+$ and is dissimilar to all other negative sample keys. Conversely, if *q* and $v_+$ are not similar, or if *q* has become similar to the key values of other negative samples, then the loss should be large, thus penalizing the model and prompting it to make parameter updates, as shown in Fig. 3.

The basic idea is to construct and maintain a dynamic dictionary for storing negative samples through a momentum update mechanism. In this framework, given a query sample and a set of key samples, the model needs to learn to match the query samples with positive samples (i.e., different views of the same image) and distinguish them from other negative samples. This process is realized by Contrastive Estimation of Neural Entropy loss^23^, which can be formulated as follows.

5 $$\begin{aligned} L _{q}=-\log \frac{exp(q\cdot v_+/\tau )}{\sum _{k=0}^K exp(q\cdot v_k/\tau ))} \end{aligned}$$

where *q* is the query sample, which is the value of the model input. $v_+$ is the key sample, which represents the sample positively correlated with the query sample *q*. $v_k$ are the other key samples, where *k* ranges from 0 to *K*, and *v* is the number of negative samples plus 1 (including the positive sample $v_+$). $\tau$: temperature coefficient to control the shape of the $q\cdot v$ distribution.

Contrastive learning calculates the gradient of the loss function with respect to each parameter through backpropagation, guiding the direction of parameter updates so that the neural network can gradually approximate the target output. Through continuous iteration and optimization, the weights of the neural network are gradually adjusted to an optimal state, enabling the network to better learn and predict the relationship between input data and target outputs. To capture the common symptoms among instances of the same type of leaf diseases and pests and the differences in symptoms between instances of different types, and to improve the recognition accuracy of the model, the CPNet model introduces supervised contrastive learning strategy.

In the training stage of the model, our goal is to learn a meaningful latent space Euclidean distance to compute the similarity between the convolutional feature maps of test images and prototypical part. The most important feature maps for classifying images are clustered (in L2-distance) around semantically similar prototypes of the images’ true classes, and the clusters that are centered on prototypes from different classes are well-separated. To achieve this goal, the CPNet uses stochastic gradient descent (SGD) to optimize the convolutional layer parameters $w_{\textrm{conv}}$ and the prototypes $\textbf{p}_m$ in the prototype layer $\mathcal {G}_{\textbf{p}}$. Where SGD uses the cross-entropy loss, the cluster loss, the separation loss and the contrastive loss, in order to reduce the total loss. The total loss can be defined as follows.

6 $$\begin{aligned} \mathscr {L}_{total}=\mathscr {C}_{ce}(\Theta )+\lambda _{1}\mathscr {C}_{\textrm{clst}}+\lambda _{2}\mathscr {S}_{\textrm{sep}}+\lambda _{3} L _{\textrm{q}} \end{aligned}$$

where $\lambda _{1}$, $\lambda _{2}$, and $\lambda _{3}$ are hyper-parameters.

The minimization of the cluster loss encourages each training image to have some feature maps that is close to at least one prototypical part of its own class, thereby enhancing the focus of the training images within the same category. The cluster loss is formulated as follows.

7 $$\begin{aligned} \mathscr {C}_{clst}=\frac{1}{N} \sum _{i=1}^{N} \min _{m: \textbf{p}_{m} \in \textbf{P}_{\textrm{y}_i}} \min _{\textbf{z} \in \text{ feature } \text{ maps } \left( f\left( \textrm{x}_{i}\right) \right) }\left\| \textbf{z}-\textbf{p}_{m}\right\| _{2}^{2} \end{aligned}$$

where *N* is the total number of inputs, $\textrm{y}_{i}$ is the label of $\textrm{x}_{i}$, and $\Vert \cdot \Vert _2^2$ is the squared L2-distance.

The minimization of the separation loss encourages every feature map of a training image to stay away from the prototypical parts not of its own class, thus increasing the distinguishability between different categories of training images. The separation loss is formulated as follows.

8 $$\begin{aligned} \mathscr {S}_{sep} = -\frac{1}{N} \sum _{i=1}^{N} \min _{m: \textbf{p}_{m} \not \in \textbf{P}_{\textrm{y}_i}} \min _{\textbf{z} \in \text{ feature } \text{ maps } \left( f\left( \textrm{x}_{i}\right) \right) }\left\| \textbf{z}-\textbf{p}_{m}\right\| _{2}^{2} \end{aligned}$$

## Results and analysis

### Experimental setup

This study was conducted on a workstation equipped with an AMD EPYC 9754 128-Core Processor and an RTX 4090D graphics card (24G VRAM), running Ubuntu 22.04 as the operating system. The experimental setup was based on a Python 3.10 environment and implemented using the PyTorch 2.1.0 deep learning framework. This framework provides efficient and flexible tools for building and training neural network models. To enhance the reproducibility of this research, all key hyperparameter configurations are listed in Table 3.

**Table 3:** Summary of training hyper-parameters in our benchmark.

Hyper-parameters	Configuration
Batch size	80
Epoch	50
Loss function	Cross entropy loss
Learning rate	0.0001
Optimizer	SGD
Input size	224 $\times$ 224 $\times$ 3
Patch size	16

### Evaluation metrics

The model is evaluated by using standard classification performance metrics. These metrics include accuracy, precision, recall, F1 Score, and Area under Receiver Operating Curve (AuC).

**Accuracy:** The accuracy rate refers to the proportion of the number of samples with accurate classification to the total number of samples. It defines the relationship between actual class values and predicted class values. The higher the accuracy value obtained by the model, the better performance is obtained. Assuming True Positive (TP) represents the number of true examples, True Negative (TN ) represents the number of true negative examples, False Negative (FN) represents the number of false negative examples and False Positive (FP) represents the number of false positive examples. The formula for the definition of accuracy is shown in Eq. (9).

9 $$\begin{aligned} A\text {ccuracy}=\frac{TP+TN}{TP+TN+FP+FN} \end{aligned}$$

**Precision:** Precision refers to the proportion of actual positive examples in all samples that are predicted to be positive.

10 $$\begin{aligned} P\text {recision}=\frac{TP}{TP+FP} \end{aligned}$$

**Recall:** Recall refers to the proportion of predicted positive examples in all samples labeled as positive examples.

11 $$\begin{aligned} R\text {e}call=\frac{TP}{TP+FN} \end{aligned}$$

**F1 score:** F1 score combines the results of precision and recall. The value ranges is [0,1], where 1 represents the best output of the model, and 0 represents the worst output result of the model. The formula of F1 score is shown in Eq. (12).

12 $$\begin{aligned} F\text {l-Score}=\frac{2*Precision*Recall}{Precision+Recall} \end{aligned}$$

**AuC:** AuC is the area covered by the receiver operating characteristic curve (ROC). The ROC is calculated by using a plot of the True Positive Rate (TPR) (refer to Eq. (13)) and the False Positive Rate (FPR) (refer to Eq. (14)).

13 $$\begin{aligned} TPR= & \frac{TP}{TP+FN} \end{aligned}$$

14 $$\begin{aligned} FPR= & \frac{FP}{FP+TN} \end{aligned}$$

In addition, the performance of the model is evaluated by using the confusion matrix and the ROC analysis plot. The confusion matrix and ROC curve indicate the credibility of the model. The higher the ROC curve in the upper left corner, the better the performance of the model. CPNet was also used to visualize prototype image classification activation maps and similarities to reveal the key rationale of the model in making classification decisions and to help researchers understand how the model works.

### Model selection and compare with other models on different datasets

The convolutional layers are the foundation of the proposed model CPNet. After 10-fold data augmentation of all datasets, three most popular models such as VGG19, ResNet152, and DenseNet161 are used to analyze their classification effects for different categories on the four dataset. Among them, VGG19 is based on the 3$\times$3 small convolutional kernels. Compared to the large convolutional kernel, it can capture the local features of the image in more detail and has less computation. ResNet152 addresses the gradient vanishing problem in deep networks by introducing residual modules and bottleneck structures, enabling the network to be deeper (up to 152 layers) and more effective in training. The features extracted from any layer of DenseNet161 can be accessed by all subsequent layers. This will cause feature reuse and reduce the amount of parameters and calculations. Due to its dense connection characteristics, DenseNet161 exhibits strong anti-overfitting capabilities and performs well even with limited training data. The three most advanced network models are used to identify diseases and pests. Its main purpose is to extract local features from each dataset in order to achieve effective classification and identification of leaf diseases and pests, facilitating in-depth comparative analysis with subsequent CPNet experimental results.

From Tables 4, 5, 6 and 7, we can see the DenseNet161 model exhibits the highest average accuracy for various categories across the four datasets, the VGG19 model performs the lowest average accuracy, and the ResNet152 model is in between. A comparison of the average accuracy for various categories across the four datasets among the three advanced models revealed that the differences in average accuracy for the Wheat Plant Diseases, Maize Leaf Disease and Pest, and Cashew Leaf Disease and Pest datasets were relatively small, generally around 2%. From the perspective of the DenseNet161 model, which has the highest average accuracy, the Cashew and Cassava Leaf Disease and Pest datasets exhibit relatively high average recognition accuracies, reaching 94.19% and 94.35% respectively. In contrast, the Maize Leaf Disease and Pest dataset has the lowest average accuracy of 85.44%. The reason for this is that there are more representative data on leaf diseases and pests for each category of the Cashew and Cassava Leaf Disease and Pest datasets, leading to higher accuracies. In the Maize Leaf Disease and Pest dataset, the visual characteristics of different disease categories (e.g., Leaf Blight and Leaf Spot) exhibit high similarity. This inter-class feature confusion significantly increases the model’s discrimination difficulty, consequently leading to suboptimal classification accuracy.

**Table 4:** Results of classification experimental use VGG19, ResNet152, and DenseNet161 on the Wheat Plant Diseases dataset.

Dataset	Category	Accuracy(%)		
		VGG19	ResNet152	DenseNet161
Wheat Plant Diseases	Healthy	88.60	92.85	95.07
	Rust	81.37	86.78	86.86
	Mildew	72.47	75.92	75.47
	Gibberellins	100	96.78	100
	Leaf Blight	93.36	93.05	97.22
	Aphid	82.23	82.94	86.47
	Mite	75.50	80.76	83.69
	Stem Fly	88.55	87.77	90.11
Average		85.26	87.10	**89.36**

**Table 5:** Results of classification experimental use VGG19, ResNet152, and DenseNet161 on the Maize Leaf Disease and Pest dataset.

Dataset	Category	Accuracy(%)		
		VGG19	ResNet152	DenseNet161
Maize Leaf Disease and Pest	Grasshopper	92.29	94.77	94.77
	Leaf Beetle	88.82	92.23	92.38
	Leaf Blight	89.99	70.34	77.42
	Leaf Spot	61.69	70.80	70.96
	Streak Virus	85.48	92.93	89.67
Average		83.65	84.21	**85.44**

**Table 6:** Results of classification experimental use VGG19, ResNet152, and DenseNet161 on the Cashew Leaf Disease and Pest dataset.

Dataset	Category	Accuracy(%)		
		VGG19	ResNet152	DenseNet161
Cashew Leaf Disease and Pest	Anthracnose	86.08	85.88	92.73
	Healthy	94.34	97.74	97.66
	Leaf Miner	90.27	92.00	87.27
	Red Rust	97.40	99.70	99.10
Average		92.17	93.83	**94.19**

**Table 7:** Results of classification experimental use VGG19, ResNet152, and DenseNet161 on the Cassava Leaf Disease and Pest dataset.

Dataset	Category	Accuracy(%)		
		VGG19	ResNet152	DenseNet161
Cassava Leaf Disease and Pest	Bacterial Blight	92.25	92.36	94.32
	Brown Spot	74.53	89.37	88.20
	Green Mite	90.05	88.94	93.39
	Healthy	92.33	96.41	98.31
	Mosaic	96.68	96.23	98.34
Average		89.14	92.66	**94.35**

In order to further evaluate the effect of CPNet, we conducted a series of comparative experiments by using VGG19, ResNet152, DenseNet161, prototypical part network, and CPNet on the test sets of four datasets. Here, Baseline represents the traditional convolutional neural networks: VGG19, ResNet152, and DenseNet161. ProtoPNet and CPNet denote the prototypical part network and the interpretable crop leaf disease and pest identification model based on prototypical part network and contrastive learning, respectively. The classification results of leaf disease and pest on four datasets using different methods are shown in Tables 8, 9, 10 and 11. From these tables, we can yield insights as follow.

In CPNet and prototypical part network, the pre-trained weights of three base models are transferred to extract input features of leaf diseases and pests, followed by classification and identification of these diseases and pests. Observe the experimental results of CPNet, prototypical part network, and traditional models, and it can be found that CPNet outperforms both prototypical part network and traditional models in terms of recognition effectiveness from various aspects, validating the effectiveness of the CPNet model.

Based on the experimental results obtained from average accuracy, precision, recall, and F1-score, Table 9 shows that the Maize Leaf Disease and Pest dataset performed poorly. The highest average accuracy on the baseline model was 85.38%, while the highest average accuracy on CPNet was 87.03%. The main reason for this is the poor classification performance of Leaf Spot and Leaf Blight, the characteristic regions of these two types of samples are scattered and their disease symptoms are extremely similar. As a result, the traditional models experience poorer feature extraction from the target regions, leading to increased recognition difficulty. However, CPNet can effectively extract local features of the maize leaf diseases and pests, leading to improved recognition accuracy. Form Tables 10 and 11, the model with CPNet using DenseNet161 as the basic feature extraction layer achieved the best recognition performance. Among them, the Cashew Leaf Disease and Pest dataset had the highest average accuracy, followed by the Cassava Leaf Disease and Pest dataset. Compared to the ProtoPNet model, the average accuracy for these two datasets improved by 1.33% and 1.02%, respectively. On the whole, through the introduction of contrastive learning, CPNet demonstrates a stronger ability to capture and understand the characteristics of crop leaf diseases and pests, resulting in an improved accuracy in identifying them.

In addition, to verify the performance stability of the model on the four datasets, using the idea of k-fold cross-validation, we processed the four datasets sequentially, with each dataset randomly divided into four parts. In each division, we use 20% of the data as a test set, while the remaining 80% is combined with the remaining three parts to form a new training set. This ensures that each part is used as a test set in one of the divisions. The CPNet model is trained on the training set using DenseNet161 as the base architecture, with validation performed on the test set. Table 12 shows the results of the 4-fold cross-validation. The average accuracies on the Wheat Plant Diseases dataset, Maize, Cashew, and Cassava Leaf Disease and Pest datasets were 92.38%, 87.03%, 96.08%, and 95.58% respectively. The experimental results demonstrate that during k-fold cross-validation across datasets, the accuracy fluctuation remains within 2%. This indicates that the CPNet model exhibits stable performance across different data subsets, showcasing strong robustness and superior generalization capabilities.

**Table 8:** Comparison of the Wheat Plant Diseases dataset classification results metrics of model selection experiment.

Method		Wheat Plant Diseases				
		Accuracy	Precision	Recall	F1 score	AuC
Baseline	VGG19	85.01	86.25	85.34	85.12	97.12
	Resnet152	87.43	87.86	87.68	87.52	97.23
	Densenet161	89.35	89.96	89.12	89.35	97.35
ProtoPNet	VGG19	88.57	88.36	88.89	89.2	97.29
	Resnet152	89.12	90.04	88.5	89.16	97.37
	Densenet161	90.74	90.49	90.47	90.77	97.54
CPNet	VGG19	89.57	89.64	89.45	89.48	97.00
	Resnet152	90.02	91.11	89.97	90.03	**97.79**
	Densenet161	**92.38**	**92.75**	**92.39**	**92.35**	97.32

**Table 9:** Comparison of the Maize Leaf Disease and Pest dataset classification results metrics of model selection experiment.

Method		Maize Leaf Disease and Pest				
		Accuracy	Precision	Recall	F1 score	AuC
Baseline	VGG19	83.95	84.35	83.12	83.88	95.35
	Resnet152	84.12	85.35	85.54	85.38	95.56
	Densenet161	85.38	87.61	85.89	86.38	95.32
ProtoPNet	VGG19	84.75	86.38	85.96	86.01	97.07
	Resnet152	84.54	86.24	85.76	85.81	95.60
	Densenet161	86.43	87.84	87.76	87.69	96.25
CPNet	VGG19	85.48	87.13	86.63	86.57	96.66
	Resnet152	86.85	88.35	87.98	88.06	97.61
	Densenet161	**87.03**	**88.58**	**88.33**	**88.21**	**97.78**

**Table 10:** Comparison of the Cashew Leaf Disease and Pest dataset classification results metrics of model selection experiment.

Method		Cashew Leaf Disease and Pest				
		Accuracy	Precision	Recall	F1 score	AuC
Baseline	VGG19	92.33	92.51	92.17	92.28	98.05
	Resnet152	93.88	93.78	93.82	93.80	98.13
	Densenet161	94.17	94.14	94.55	94.22	98.18
ProtoPNet	VGG19	93.25	93.18	93.45	93.30	98.23
	Resnet152	94.00	93.92	93.29	94.05	98.05
	Densenet161	94.75	94.6	94.96	94.75	98.51
CPNet	VGG19	94.83	94.76	94.80	94.98	98.36
	Resnet152	95.15	95.16	95.45	95.28	98.28
	Densenet161	**96.08**	**96.07**	**96.19**	**96.13**	**98.59**

**Table 11:** Comparison of the Cassava Leaf Disease and Pest dataset classification results metrics of model selection experiment.

Method		Cassava Leaf Disease and Pest				
		Accuracy	Precision	Recall	F1 score	AuC
Baseline	VGG19	89.13	89.98	89.42	89.46	98.06
	Resnet152	92.46	92.69	92.60	92.64	98.14
	Densenet161	94.31	94.40	95.07	94.69	98.03
ProtoPNet	VGG19	91.26	91.90	91.57	91.71	98.28
	Resnet152	93.66	93.96	94.76	94.31	98.13
	Densenet161	94.56	94.82	95.25	94.63	98.43
CPNet	VGG19	94.53	95.20	94.62	94.90	98.25
	Resnet152	95.02	95.25	95.19	95.21	98.18
	Densenet161	**95.58**	**95.55**	**95.38**	**95.46**	**98.74**

**Table 12:** CPNet-Densenet161 test results based on k-fold cross-validation.

	Accuracy			
No of Fold	Wheat Plant Diseases	Maize Leaf Disease and Pest	Cashew Leaf Disease and Pest	Cassava Leaf Disease and Pest
1-fold	91.85	86.52	95.78	95.12
2-fold	92.16	87.49	96.26	95.82
3-fold	92.82	87.25	96.38	95.89
4-fold	92.72	86.88	95.93	95.52
**Average**	92.38	87.03	96.08	95.58

### CPNet classification experiments

**Figure 4:**
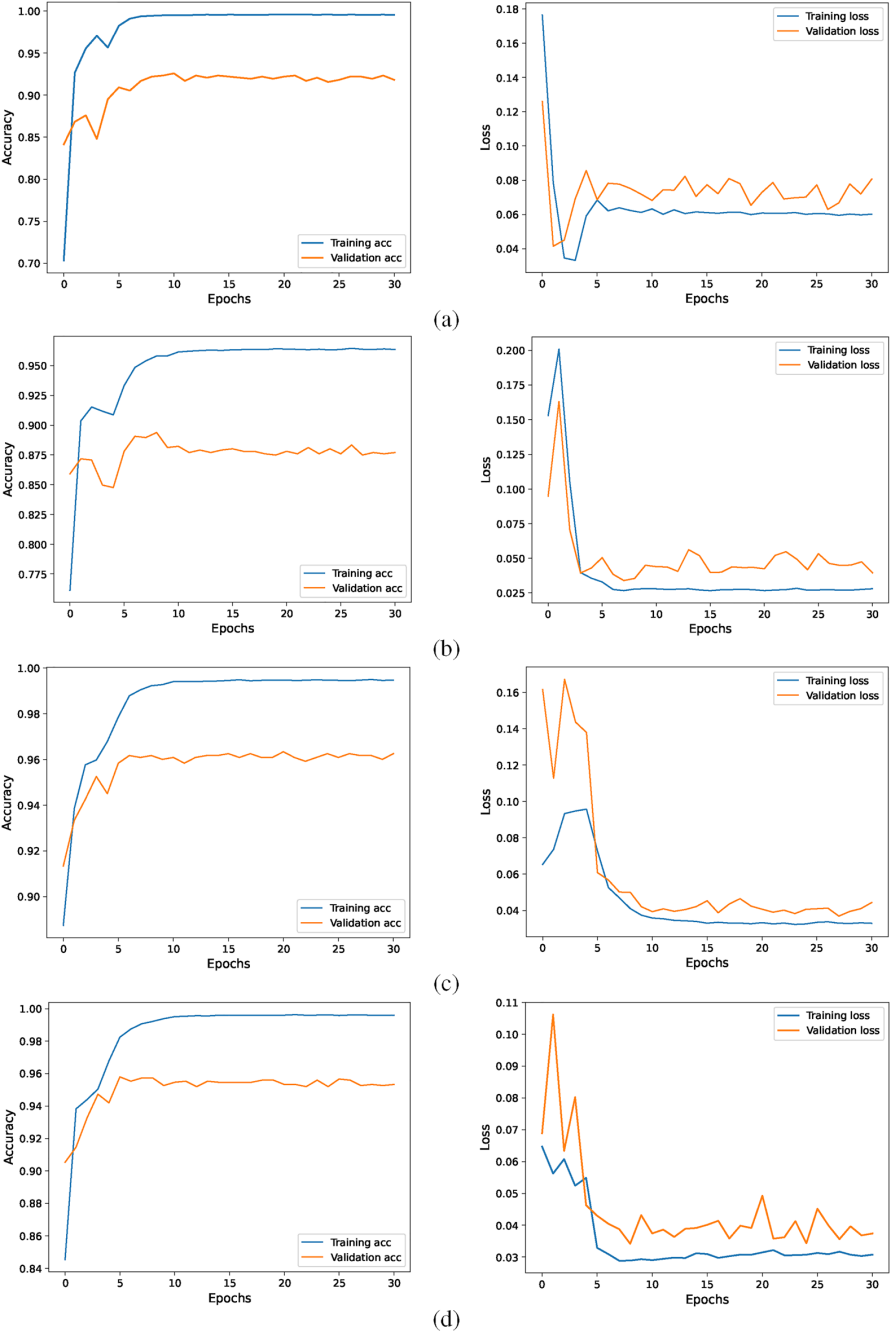
The accuracy and loss curves of CPNet on four datasets: (**a**) Wheat Plant Diseases, (**b**) Maize Leaf Disease and Pest, (**c**) Cashew Leaf Disease and Pest, and (**d**) Cassava Leaf Disease and Pest.

The training and testing accuracies of all datasets are shown in the left four accuracy graphs in Fig. 4. During the model training process, the accuracy of the model initially increases rapidly and then begins to stabilize after a certain number of epochs, ultimately achieving optimal performance. The two datasets with the highest accuracy are Cashew and Cassava Leaf Disease and Pest datasets. The overall trend of accuracy across the four datasets is upwards and convergent, indicating that the model gradually learns to distinguish between samples of different categories and recognize the image features of various diseases and pests in the four datasets.

After each sample passes through the CPNet model, it generates a predicted value, and the difference between this predicted value and the true value becomes the loss. The loss functions produced by our model are presented in the four loss graphs in the right column of Fig. 4. For the four datasets of the Wheat Plant Diseases, Maize Leaf Disease and Pest, Cashew Leaf Disease and Pest, and Cassava Leaf Disease and Pest, the loss curves gradually converge. However, for the Cassava Leaf Disease and Pest dataset, there are fluctuations in the convergence of the loss, indicating that the learning process encounters a bottleneck. The reasons for this difference may include the imbalance of samples in certain classes within the dataset or the high similarity in texture between different types of diseases, which can result in misclassification of disease and pest samples and fluctuations in the loss effect.

**Figure 5:**
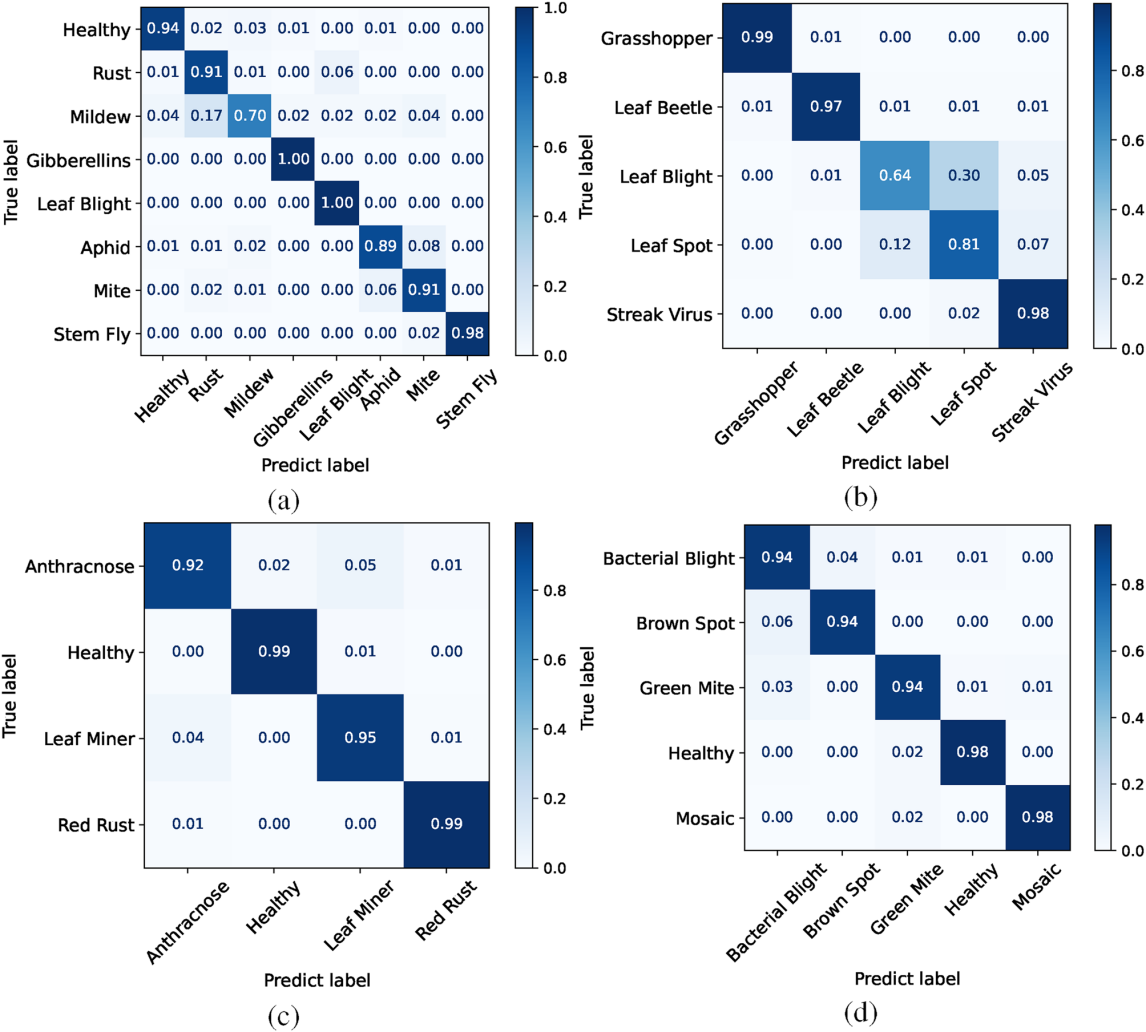
The confusion matrix of CPNet on the four datasets: (**a**) Wheat Plant Diseases, (**b**) Maize Leaf Disease and Pest, (**c**) Cashew Leaf Disease and Pest, and (**d**) Cassava Leaf Disease and Pest.

The confusion matrix is one of the evaluation metrics for model classification and recognition. Each row of the confusion matrix represents the true class of the data, while each column represents the predicted class. By using CPNet, confusion matrices were created for the test sets of the four datasets, as shown in Fig. 5. It can be observed that CPNet performs well on Fig. 5c,d, with the classification accuracy of each pest and disease category for the Cashew Leaf Disease and Pest dataset exceeding 94%. Form Fig. 5a, we can see that the classification of Gibberellins and Leaf Blight in the wheat dataset is highly accurate, achieving 100% accuracy. However, Mildew exhibits significant asymmetric misclassification, with only a 70% accuracy, a 17% probability of being misclassified as Rust, and a reverse misclassification rate of just 1%. This asymmetric misclassification pattern stems from the high visual similarity between the early symptoms of Mildew and Rust, coupled with an asymmetric distribution of the decision boundaries in the feature space. Form Fig. 5b, the classification accuracy for Leaf Blight is the lowest at 64%, with 30% of the test images being misclassified as Leaf Spot due to the similarity in image features between the two diseases, making them difficult to distinguish. Therefore, it can be observed that leaf diseases and pests with smaller local differences in image features are more likely to be misclassified, and the local similarity of visual features between categories is positively correlated with the model’s misclassification rate. Through the confusion matrices, we clearly present the classification results of CPNet on the four datasets in a matrix form, helping us understand the model’s performance across different categories.

**Figure 6:**
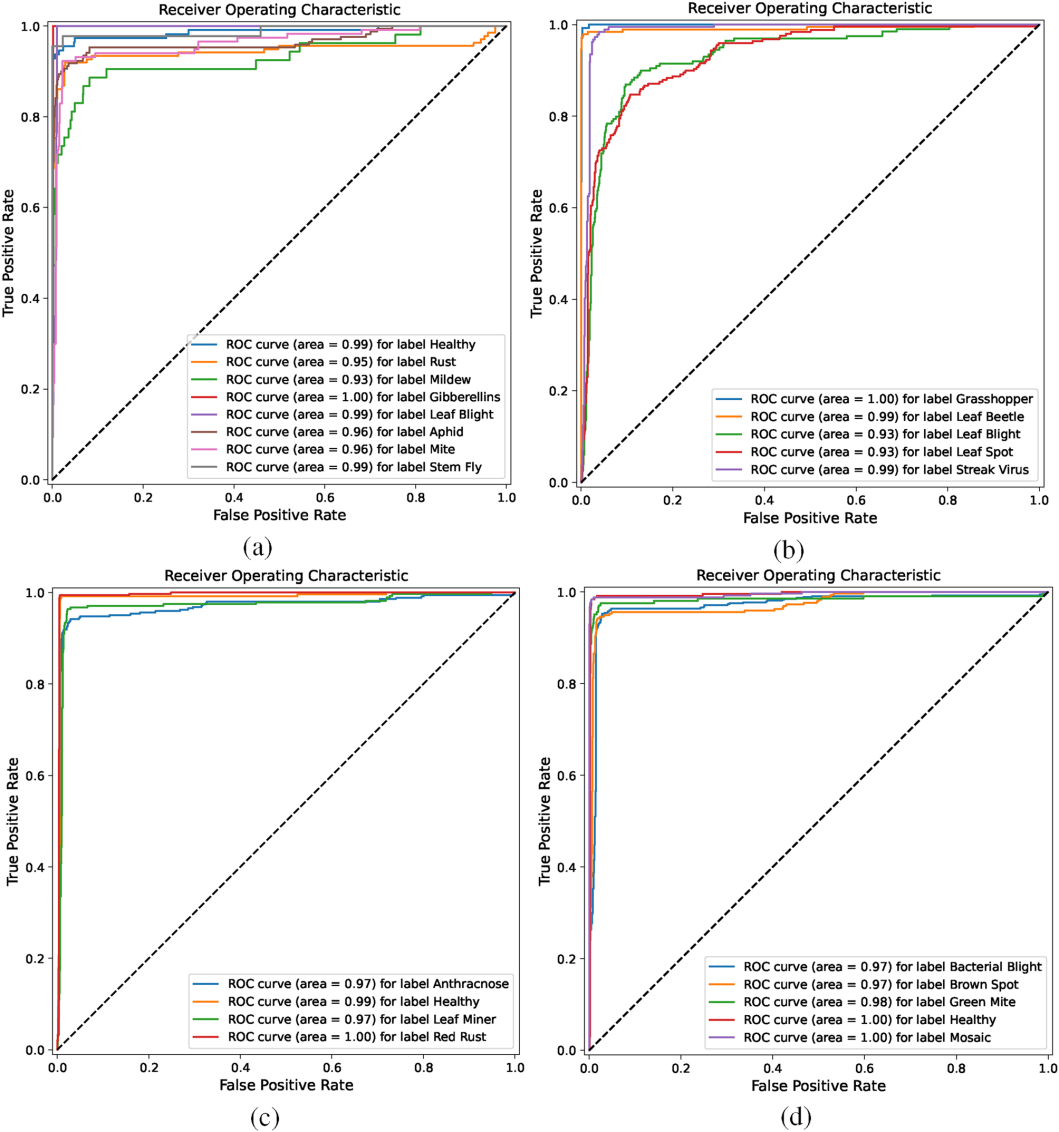
The ROC of CPNet on the four datasets:  (**a**) Wheat Plant Diseases, (**b**) Maize Leaf Disease and Pest, (**c**) Cashew Leaf Disease and Pest, and (**d**) Cassava Leaf Disease and Pest.

The ROC curve is used to evaluate and compare the performance of classification models. In the coordinate system, the vertical axis represents the TPR (True Positive Rate/Hit Rate/Recall), with a maximum value of 1, and the horizontal axis represents the FPR (False Positive Rate/Fall-Out), also with a maximum value of 1. The dashed line serves as the baseline (minimum standard), while the colored curves are the ROC curves. The further the ROC curve is from the baseline, the better the prediction performance of the model. By plotting the relationship between the True Positive Rate (TPR) and False Positive Rate (FPR) under different thresholds, the ROC curve provides an intuitive display of the model’s predictive ability and measures the model’s ability to distinguish between positive and negative samples. From Fig. 6c,d, we can see CPNet performs best on the Cashew and Cassava Leaf Disease and Pest datasets, with ROC curves close to the top-left corner, indicating high model prediction accuracy and excellent ability to distinguish between positive and negative samples. However, in Fig. 6b, the distinction between positive and negative samples for Leaf Blight and Leaf Spot disease in the Maize Leaf Disease and Pest dataset is poor. This is due to the extreme similarity in local features between these two diseases and the scattered distribution of affected areas, which affects the model’s learning effectiveness. Therefore, when the model encounters leaf disease feature extraction where the infected areas are extremely similar and the disease characteristics are scattered, its performance can be impacted, leading to poor recognition results.

### CPNet interpretability analysis

**Figure 7:**
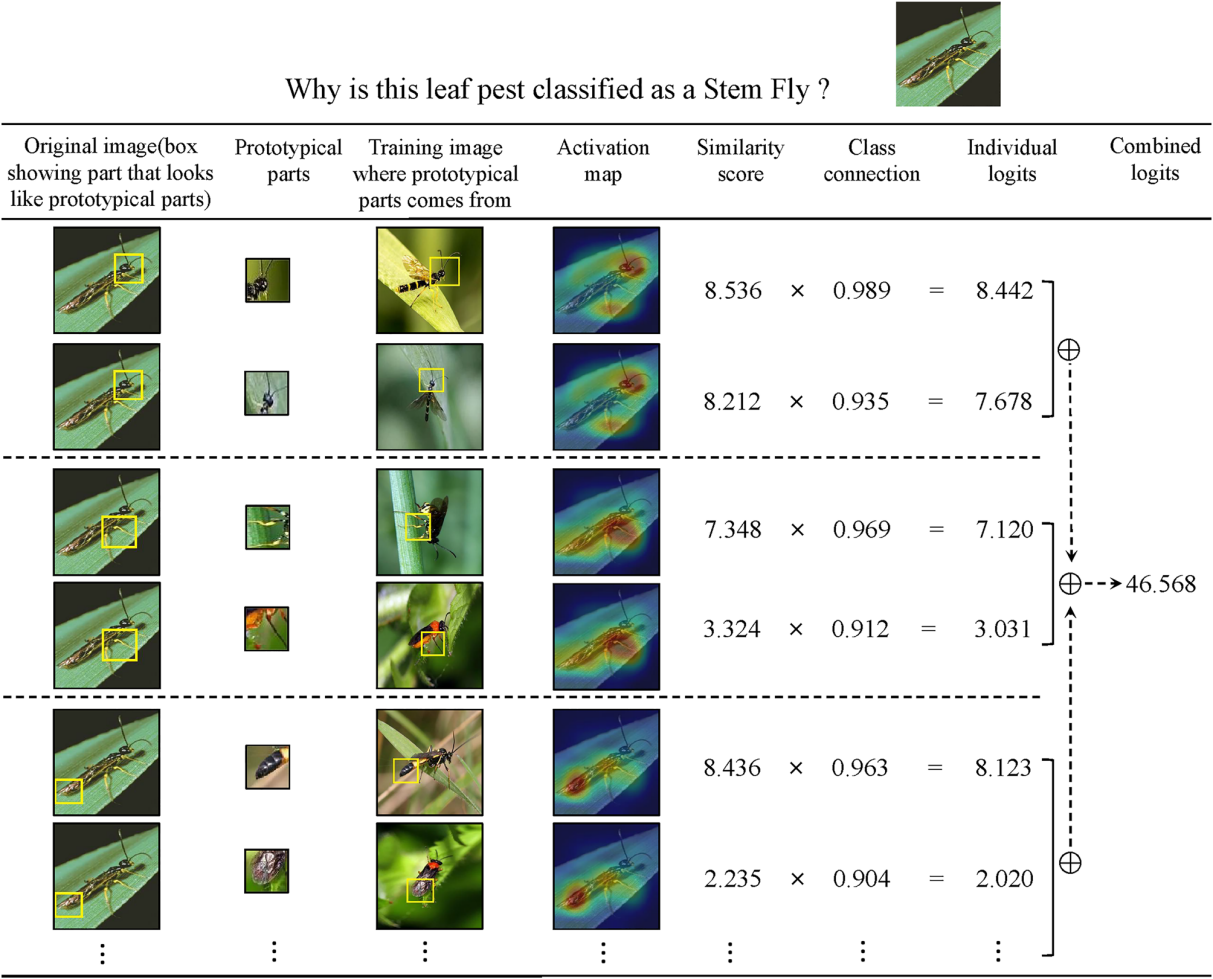
The reasoning process of CPNet in deciding the species of the Stem Fly.

**Figure 8:**
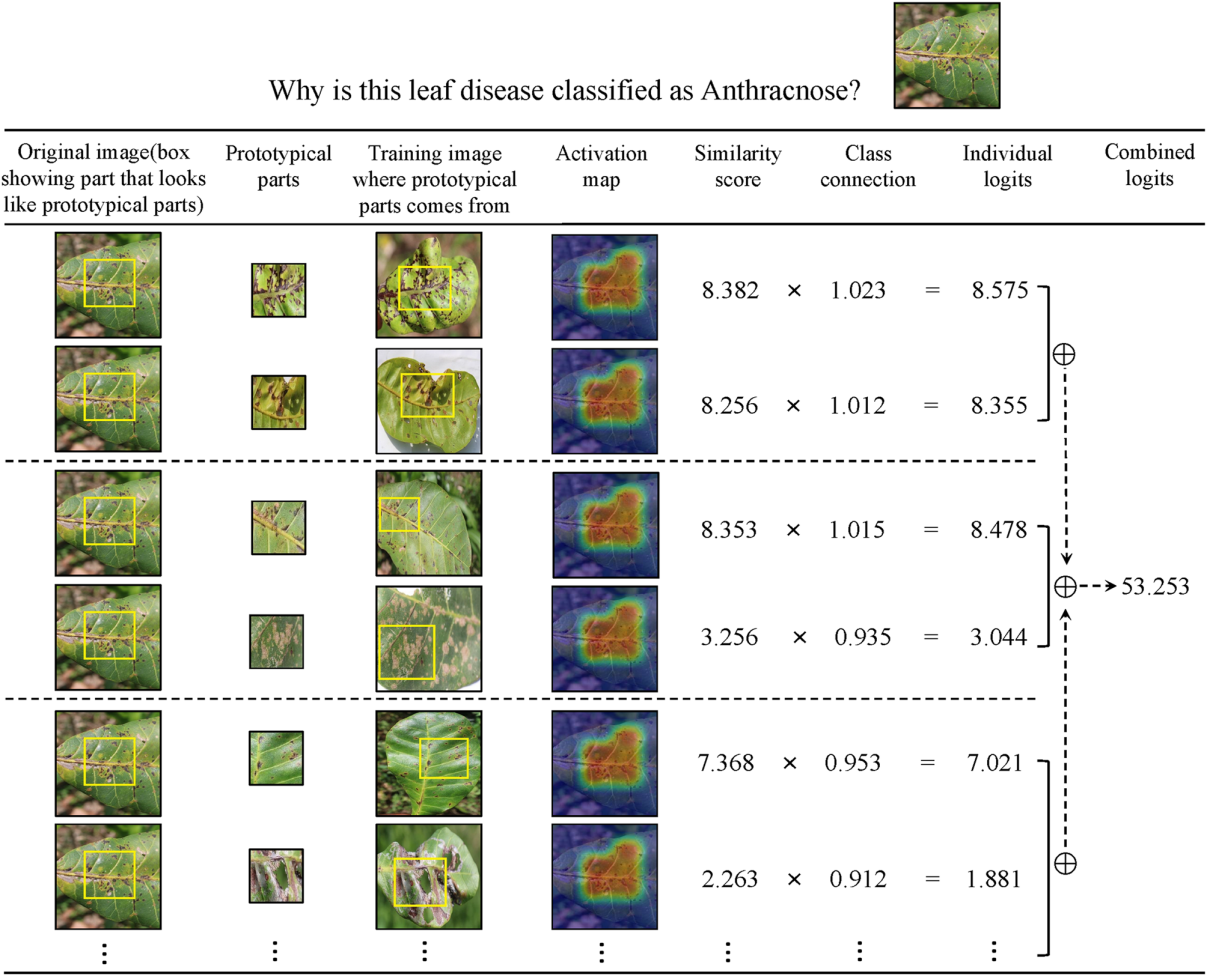
The reasoning process of CPNet in deciding the species of the Anthracnose.

As an interpretable model, CPNet can not only predict the categories of leaf diseases and pests but also identify the key regions that influence the model’s decisions. This enables interpretable image classification and recognition of crop leaf diseases and pests. Figure 7 shows the reasoning process by which CPNet makes a classification decision for the test image of the Stem Fly directly above the figure. Given a test image $\textrm{x}$, the model compares its latent features $f(\textrm{x})$ to a learned prototypical parts. For each class *t*, our network tries to find evidence that $\textrm{x}$ belongs to class *t* by comparing the convolutional feature maps of $\textrm{x}$ with each learned prototypical part $\textbf{p}_{\mathfrak {m}}$ of class *t*. For example, in Fig. 7, CPNet attempts to find evidence for the class of Stem Fly by comparing the latent feature map of the images to each prototypical part of the class (shown in the “Prototypical parts” column). This comparison produces a similarity scores map for each prototypical part, which is up-sampled and overlaid on the original image to see which part of a given image is activated by the prototypical part. As shown in the “Activation map” column of Fig. 7, the first type of prototypical part (the first two rows of prototypical part maps) of the Stem Fly class is most strongly activated on the head of the test fly, and the second type of prototypical part (the middle two rows of prototypical part maps) is most strongly activated on the limbs. The most strongly activated image feature map in the test image corresponding to each prototypical part (in the “Original image” column) is marked with a bounding box by CPNet - This is the feature map of the image that CPNet thinks is the most similar to the corresponding prototypical part. In this case, CPNet has found a high similarity between the head of the fly and the prototypical head of the Stem Fly in the given input image (similarity scores of 8.536 and 8.212, respectively), as well as between the limbs and the limbs of the prototypical part of the first row (similarity scores of 7.348). These similarity scores are weighted and added together, which will result in a final score for the fly belonging to this class, and the reasoning process is similar for all other classes (e.g., Fig. 8). The network ultimately correctly classifies the fly in the input image as a Stem Fly.

The entire reasoning process demonstrates how our CPNet predicts and classifies previously unseen images of leaf diseases and pests, proving that our model can not only effectively identify diseases and pests on agricultural leaves but also explain the key basis for the model’s classification decisions. This key basis is the prototypical part in the training images, which can assist researchers in understanding the working principles of the model. In this way, our model is interpretable due to its transparent reasoning process when making predictions.

## Conclusion

In current research, to effectively extract target local features from images of crop leaf diseases and pests, most researchers employ deep learning models to extract disease and pest features on leaves for recognition. However, these models are often seen as “black box”, making it difficult to explain the basis for their specific decisions, and the model outputs are in an uninterpretable state, casting doubt on the credibility of the models. To address this issue, we propose an interpretable identification model for crop leaf diseases and pests based on prototypical part network and contrastive learning. We conducted experiments on the four public datasets, and the results show that this network outperforms existing traditional models and provides interpretability for the model. Since the CPNet model is a spatially rigid prototypes, it cannot explicitly explain the scattered distribution of affected areas in crop leaf diseases and pests. In future work, we will explore the interpretable recognition of the scattered distribution of affected areas in crop leaf diseases and pests through spatially flexible prototypes.

## Data Availability

The data used in this study is publicly available at the following link: https://data.mendeley.com/datasets/bwh3zbpkpv/1 and https://www.kaggle.com/datasets/kushagra3204/wheat-plant-diseases.
